# Visualization of Ion|Surface Binding and In Situ Evaluation of Surface Interaction Free Energies via Competitive Adsorption Isotherms

**DOI:** 10.1021/acsphyschemau.1c00012

**Published:** 2021-08-23

**Authors:** Pierluigi Bilotto, Alexander M. Imre, Dominik Dworschak, Laura L. E. Mears, Markus Valtiner

**Affiliations:** Institute of Applied Physics, Applied Interface Physics, Vienna University of Technology, 1040 Vienna, Austria

**Keywords:** adhesion, competition, ion free energy

## Abstract

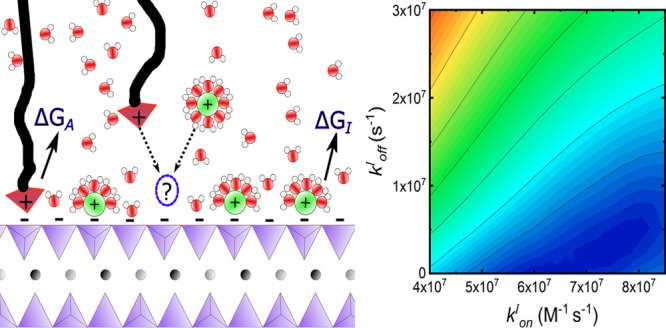

Function and properties at biologic as well as technological interfaces are controlled by a complex and concerted competition of specific and unspecific binding with ions and water in the electrolyte. It is not possible to date to directly estimate by experiment the interfacial binding energies of involved species in a consistent approach, thus limiting our understanding of how interactions in complex (physiologic) media are moderated. Here, we employ a model system utilizing polymers with end grafted amines interacting with a negatively charged mica surface. We measure interaction forces as a function of the molecule density and ion concentration in NaCl solutions. The measured adhesion decreases by about 90%, from 0.01 to 1 M electrolyte concentration. We further demonstrate by molecular resolution imaging how ions increasingly populate the binding surface at elevated concentrations, and are effectively competing with the functional group for a binding site. We demonstrate that a competing Langmuir isotherm model can describe this concentration-dependent competition. Further, based on this model we can quantitatively estimate ion binding energies, as well as binding energy relationships at a complex solid|liquid interface. Our approach enables the extraction of thermodynamic interaction energies and kinetic parameters of ionic species during monolayer level interactions at a solid|liquid interface, which to-date is impossible with other techniques.

## Introduction

All active systems that are subject to change, motion, or flow of matter (i.e., all biologic systems, and all mechanical systems) are governed by molecular level interactions that drive and steer the way in which macroscopic structures develop, evolve, adapt, and age. Consequently, the study of molecular interactions is a shared and fundamental interest in seemingly unrelated fields, such as biophysics^1^ and adhesion,^2^ corrosion science,^3^ and stem cell research,^4^ or electro-osmosis in ion channels.^5^ In essence, competing molecular interactions, such as competitions of different specific and unspecific bonds, drive subtle molecular balances and equilibria in the complex machinery of life and in technology.

In the last decades, the surface forces apparatus (SFA) and atomic force microscope (AFM) have been extensively used to probe a variety of interactions to establish their nanomechanical and dynamic properties. This includes studies on biofouling of marine fauna,^6−8^ receptor–ligand interactions,^9,10^ engineered lipid bilayer membranes,^11,12^ and polymers investigated for different density, electrolyte, or pH conditions.^13,14^ Further, many studies on specific binding systems have been performed in order to establish an understanding of interfacial interactions across the full range of length and energy scales, thus bridging the gaps between molecular scale interactions and macroscopic properties.^15−17^ Still, we lack a detailed understanding of how molecular level competition and interplay impact macroscopic interactions in complex media such as physiological solutions containing a complex mixture of ions and water,^18^ as well as functional molecules.^19,20^ Generating a detailed molecular understanding of complex, simultaneous interactions at reactive and/or dynamic solid|fluid interfaces is a challenge across disciplines, and has intrigued researchers for decades.^21−25^ Whether it is, for example, in medical adhesives, friction of articular cartilage,^26^ or the adhesion of organisms in seawater,^24^ complex macroscopic properties at crowded biologic solid|liquid interfaces are mediated by large numbers of individual nanoscale interactions;^27^ namely similar or dissimilar molecule/molecule and molecule/surface interactions, surface–dipole interactions,^28^ or the competing interactions with ions and water.^29^ The structure of interfacial bound species such as strongly binding water can, for example, produce surfaces that are highly resistant to protein adsorption and fouling.^30^

It was further demonstrated that exactly this complex competition and molecular structuring at interfaces are central to a multitude of interfacial phenomena, such as membrane transport,^31^ membrane conductance,^32,33^ cellular adhesion,^34^ and adhesion regulation in the marine environment.^35^ It has been speculated that subtle concentration changes may play a role in activating/deactivating enzymatic catalysis in biologic systems. Further, competitive interactions are the foundations of different adhesive and electrolyte related technologies, such as the generation of stable biomaterial for dental reconstruction^36^ or adhesive tunable hydrogels for ultracold environments^37^ and electrically programmable adhesive hydrogels.^38^ As such, how hydration and ion effects alter molecular interactions is central to a large range of processes.

**Figure 1:**
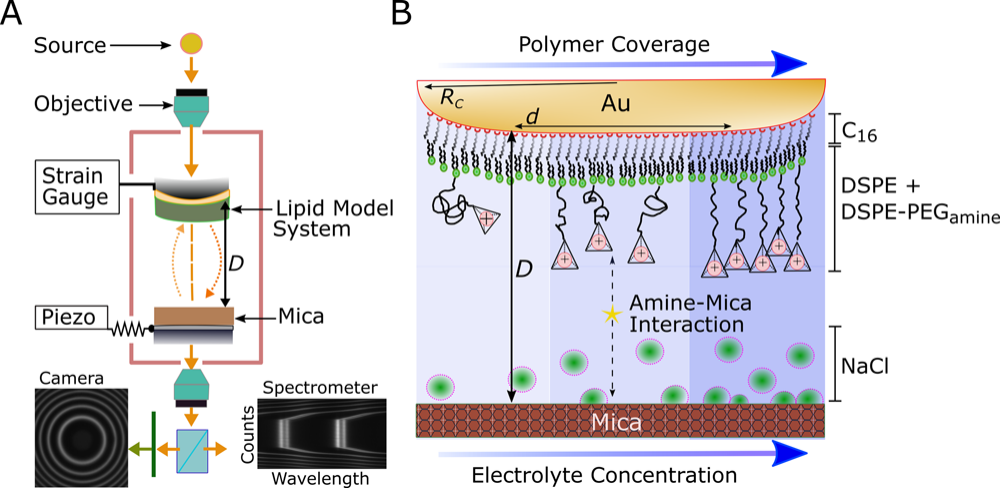
(A) Schematic of the SFA setup. The colored lines indicate the optical path (*c.f.*Methods and Materials for details). (B) Sketch of the lipid model system (LMS). From top to bottom: the gold substrate was template stripped on a quartz disk with curvature *R*_*C*_. A layer of C_16_ is chemically adsorbed to ensure stability. The outer-leaf of the LMS is a mixture of DSPE and DSPE-PEG_amine_ at a set percentage/coverage Γ. Likewise, the sodium chloride concentration varies in a range from 0.01 to 1 M.

In this work, and shown in Figure 1, we employ the SFA to investigate the specific electrostatic interaction between a positively charged amine functionality (varied in density during the experiments) and a negatively charged mica surface. Specifically, we examine how interaction forces are affected by the electrolyte concentration. The increasing concentration induces a competition between the ions of the electrolyte and the amines for the interfacial binding sites. On the basis of a kinetic model using two competing Langmuir adsorption isotherms we can estimate ion/surface interaction energies from the experimentally recorded interaction force measurements, demonstrating a path for a comprehensive combined experimental and modeling approach.

## Results and Discussion

**Figure 2:**
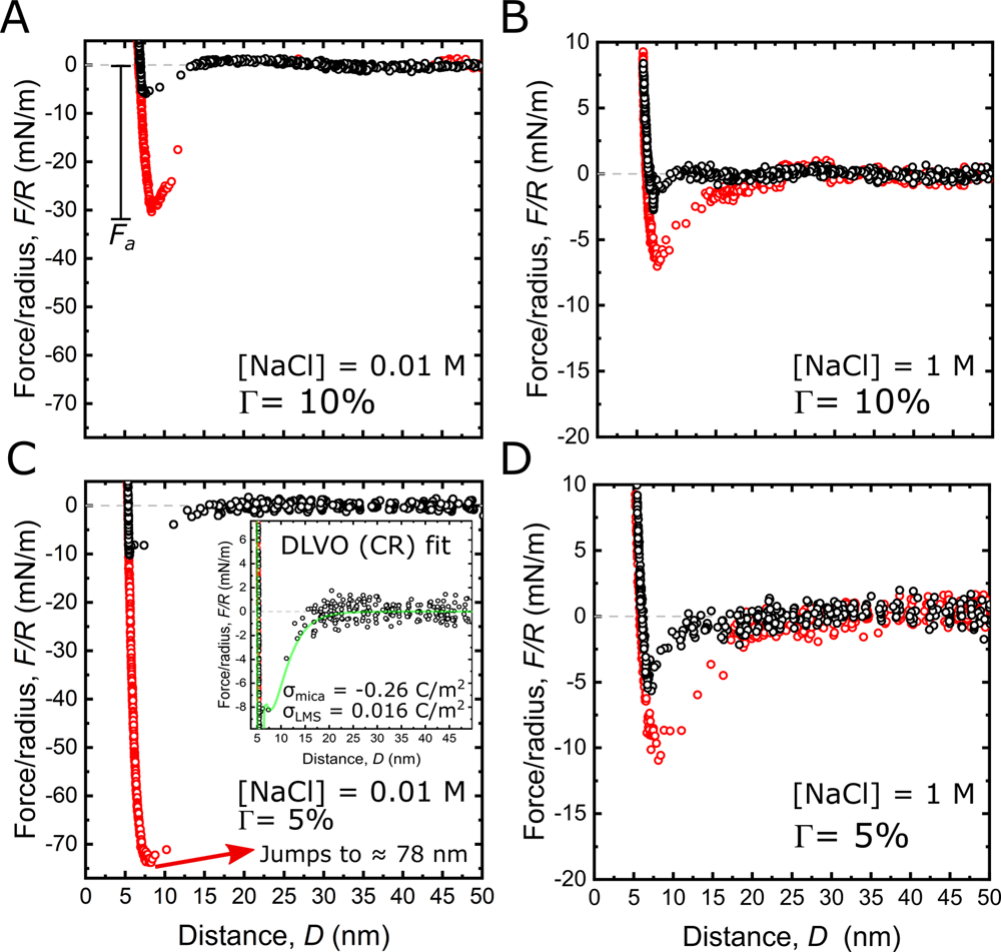
Force profiles of LMS in different configurations. In panels A and B LMS with coverage Γ = 10% is tested in 0.01 and 1 M of sodium chloride. In panels C and D LMS with a coverage Γ = 5% is tested in similar electrolyte conditions. In black we indicate the surface approach and in red the surface retraction. The minimum of each red curve defines the adhesion force *F*_a_. *D* = 0 is defined as the dry mica|gold contact. The inset in panel C shows in green a charge regulated DLVO fit of our data. Additional details are reported in the text.

Figure 2 shows selected examples of force versus distance characteristics obtained as a function of salt concentration and amine-terminated polymer coverage Γ. During the approach of one surface to the other (black markers), a mechanical instability (*jumps-in* to contact) is observed at a distance *D* ∼ 15 nm close to the fully extended contour length of the polymer tether (*L*_*c*_ = 12.5 nm), plus the thickness of the inner monolayer of C_16_ and the outer lipid layer.^11^ The jump into contact is mediated by the specific intermolecular interaction between the positively charged amines and the negatively charged mica binding sites, which indicate a shorter range at higher ion concentrations due to the expected screening effect. This *jump in* brings the surfaces into a strong adhesive contact, with the brush compressed to about 60–70% of its contour length at *D* ∼ 7–8 nm as expected for a brush with 5–10% coverage.^39^

During the retraction of one surface from the other (red markers), an adhesive hysteresis, with an approximately 3–4 nm molecular extension and consequent *jumps-out* from contact is recorded, separating the surfaces to a large distance at zero force. The observed molecular extension to about 80–90% of the contour length reflects the stretching of specifically bound polymers—via the amine|mica interaction—as well as hydration of the contact under the increasing tensile load acting on the formed adhesive contact.

In Figure 2A, we indicate the maximum of adhesive force, *F*_*a*_, which is important for further discussion. These instability phenomena, where surfaces jump apart (*jumps-out*), are typical of SFA measurements^40^ and they are observed, with excellent reproducibility, over up to 15 consecutive force versus distance characteristics, confirming the quality and stability of the lipid model system. Moreover, as an inset of Figure 2C, we provide a DLVO fit for asymmetric surfaces in the limit of the charge regulation approach, tested on similar systems in our previous work.^11^ Mica and lipid model system surface charges σ are expected to be −0.3 and 0.02 C/m^2^, respectively.^40^ Our fit presents σ_mica_ = −0.26 ± 0.01 and σ_LMS_ = 0.016 ± 0.002 C/m^2^, for the couple of regulator parameters *p*_1_ = 0.3 and *p*_2_ = 0.95 at *C* = 0.01 M for a van der Waals plane of origin located at *D*_VdW_ = 5.6 nm.^11^

As shown in the four panels of Figure 2, the magnitude of *jumps-in* minima and the adhesion force *F*_a_ vary significantly with the lipid composition (polymer coverage Γ), as well as with the environment (electrolyte concentration [NaCl]). Consequently, *F*_*a*_ is a function of Γ and [NaCl]; therefore, we investigated five different polymer coverages (i.e., amine coverages), each tested in six different electrolyte concentrations to unravel their influence on the amine|mica interaction.

**Figure 3:**
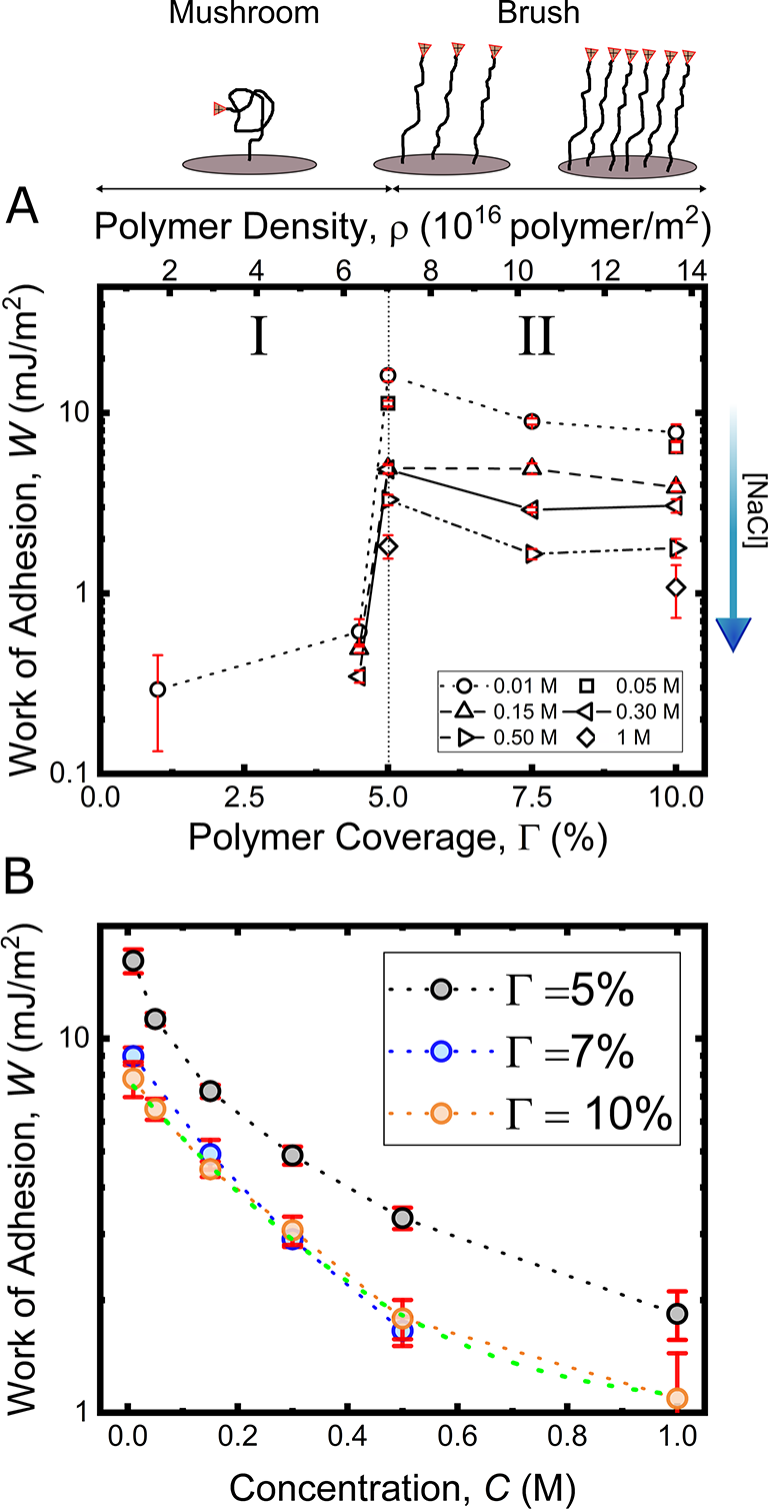
(A) Semilog plot of the averaged work of adhesion *W* as a function of the polymer coverage Γ and the electrolyte concentration *C*. On top, the polymer density axis ρ together with self-explanatory sketch of polymer configurations. The variation of ρ generates two regions: the mushroom (I) and the brush (II) regimes. (B) Semilog plot of the work of adhesion as a function of the electrolyte *C* at different polymer coverages Γ.

The adhesion force *F*_a_ can be further converted to work of adhesion by applying the Derjarguin Approximation in the limit of the JKR model.^40,41^ In Figure 3, we present a semilog plot of the experimental work of adhesion *W*, against the polymer coverage Γ at the bottom *x*-axis and the polymer density ρ (on top). Each data point is the average of the work calculated from at least five force runs, with the error bars defined as the standard deviation. The polymeric part of our lipid model system, consisting of a PEG(2000) chain, exhibits a radius of gyration *R*_G_ = 1.7 nm.^42^ The polymer density ρ is defined during the sample preparation, and from it we derive the distance between grafting points *s*. Thus, the polymer density was varied from a mushroom (*s* > 2*R*_G_) to a brush (*s* < 2*R*_G_) regime (indicated by region I and region II, respectively, and shown in the schematic above the plot).^43,44^ The data reveal a maximum of the measured interaction force at the transition from the mushroom to the brush regime.

The generally lower *W* that we observe in region I is consistent with the assertion that the adhesion is driven by the amine|mica interaction. Fewer amines are available owing to the low polymer density, that is, the configuration that the polymer takes in the solvent. However, the amines are also hidden within a tangle of soft material, away from the binding site in the mushroom structure. There, it is energetically unfavorable for the polymer to leave the mushroom configuration and orient the amine toward the mica binding sites. Hence, it is more likely that the backbone of the polymers participate in the interaction with the probing surface, generating a steric repulsion, which overpowers the amine|mica bond, lowering the overall measured adhesion. As a side note, at very low polymer concentrations the mushroom repulsion breaks down, leading to a rapid increase in the underlying van der Waals interactions. Consequently the adhesion increases significantly compared to when the polymer remained in the contact area, in line with our previous observations of the mica|bilayer interface.^11^

In line with this argument a sudden increase of about one order of magnitude and maximum of adhesion is observed at the transition from the mushroom into the brush regime. At the transition (Γ = 5%), the polymers are forced into the brush regime during deposition, making the amines available at the surface under no structural constraints. Therefore, when the polymer-covered lipid layer is facing the mica, no additional energy is needed to stretch the polymers to face them toward the binding surface, explaining the sudden increase of the adhesion in the brush regime.

Increasing the coverage Γ further into the brush regime again results in a now gradual depletion in *W* (right side of Figure 3A) by about 30%.

It is our understanding that the maximum of adhesion at the mushroom—brush boundary is hence due to a combination of entropic and background effects. First, again following an entropic argument the lateral interaction in a more and more crowded brush of polymers, reduces the mobility of the single chains during their interaction with mica binding sites. Consequently, the cooperation of polymeric steric effects participating in the amine|mica interaction could result in damping the measured adhesion, by lowering the configurational entropy of the possible bonding scenarios. Second, at lower densities a slightly smaller (about 1 nm) hard wall distance, and hence an increasing influence of background van der Waals interactions, are likely to take place. As a result, the data at the highest compression is approaching a situation where the interfacial amine|mica bonding is the dominating contribution to the interaction free energy/work of adhesion. Given the binding site densities, we can further estimate that even at 10% coverage a considerable excess of 15 mica binding sites are available for one amine.

On the right side of Figure 3A, a blue arrow indicates the adhesion decreasing with the electrolyte concentration. In detail Figure 3B shows how the increment in concentration results in an exponential decay of the work of adhesion measured at each coverage Γ (e.g., see indicated exponential trend in green). This suggests a competition of ions and amines for the negatively charged binding sites on mica. Following the argument above, we focus the further analysis on the situation at 10% coverage, in which the amine|mica interaction is the dominating contribution to the measured interaction free energy/work of adhesion

We now show that this competition can be described semiquantitatively in terms of two competing Langmuir adsorption isotherms. One for the ions and one for the amines adsorbing on the mica binding sites in a competitive equilibrium that depends on the concentrations of the involved species. In the Methods and Materials section, we provide the details of how to describe a competition in terms of the population of all the species active in the chemical equilibrium (see eq 3), using the law of mass action. Here we show this for the 10% coverage as an exemplary case.

Briefly, we establish a system of differential equations, describing two competing Langmuir isotherms, needed to simulate the amine|mica bond formation (see eq 5), which are then solved numerically. In terms of the adhesion promotion by the amines, we are interested in estimating the binding density *B*/*A*, that is, the ratio between the total number of bonds formed  (bound amines), and the total amount of amines available for bond formation (bound and unbound) . This quantity is a direct outcome of the numeric solution of the set of differential equations. Precisely, it is the ratio of simulated concentrations *x*_3_ and *x*_1_ after full equilibration of the competing isotherms as a function of the increase of the ion concentration (see Methods and Materials section).

To compare the simulated *B*/*A* with the measured SFA work of adhesion we introduce a “simulated work of adhesion, *W*_*s*_”. Using the polymer, that is, the amine density at 10% coverage, we can estimate the amine|mica bond energy *W*_0_ based on dividing the measured work of adhesion at 0.01 M, by the polymer density ρ (polymer/m^2^), defining an upper bound for the interaction free energy. This defines the simulated interaction free energy in terms of  mJ/m^2^, with . Therefore, the equilibrium constant estimated from eq 4 using *W*_0_ is *K* = 1.15 × 10^–6^, or p*K* = 5.93. This is a reasonable value for an amine functionality, which agrees well with that in the literature.^45−48^

**Figure 4:**
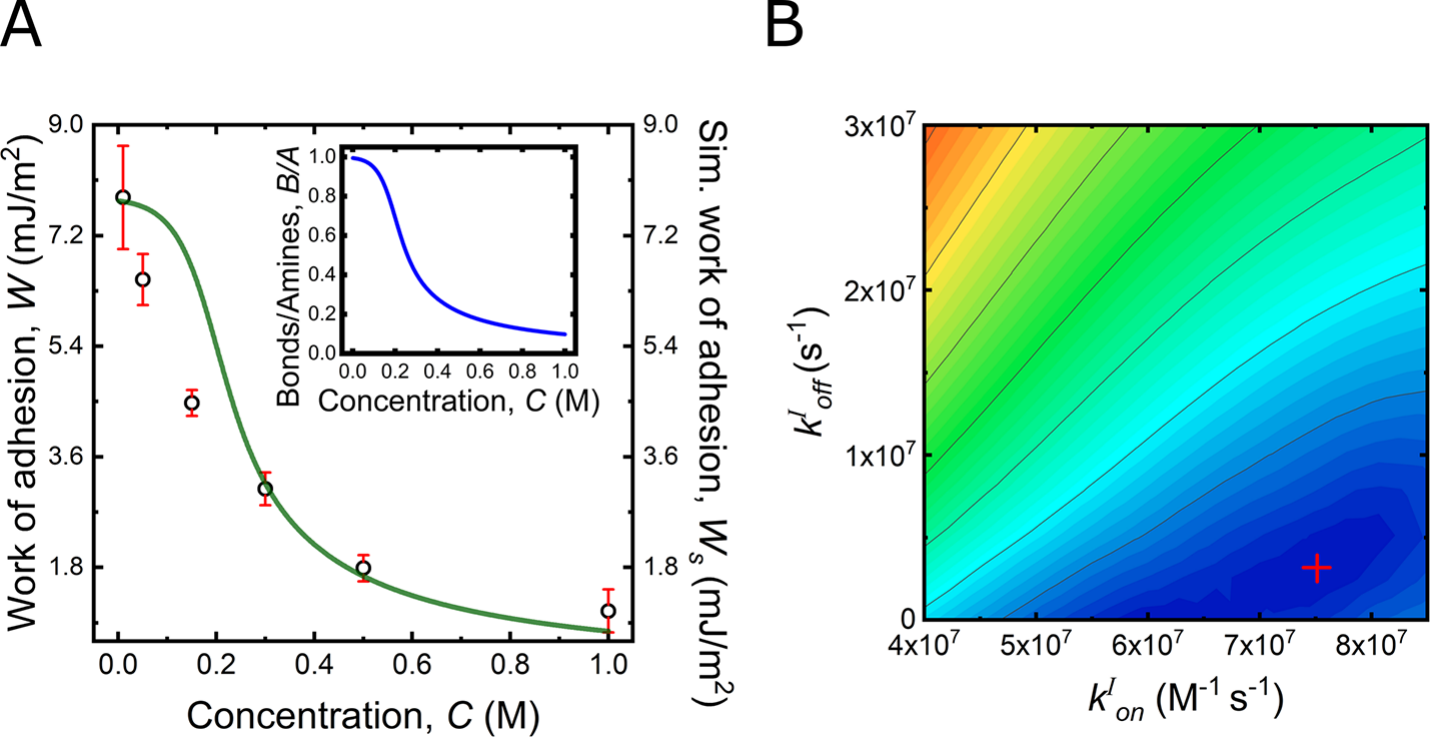
Adhesion and ion competition. (A) Comparison between experimental (left axis, black circles) and simulated (right axis, green line) work of adhesion plotted against the electrolyte concentration for Γ = 10%. The inset shows the binding density *B*/*A* as a function of the electrolyte concentration. (B) RMSD map as a function of the rate constants for ions *k*_on_^I^ and *k*_off_^I^. The color scale represents the RMSD value from blue to red, lowest to highest, respectively. The red cross indicates the estimated ionic rate constants.

In Figure 4A we now show on the right *W*_s_ and on the left the measured scale as a function of the electrolyte concentration *C*, for Γ = 10%. The experimental work *W* decays exponentially with the concentration. The simulated curve follows well the exponential trend, with deviations in a concentration window between 0.05 to 0.2 M.

Hence, overall the competing Langmuir isotherm model can predict the observed experimental trend very well, while the simulation warrants a deeper discussion. In detail, this model is to be considered as semiquantitative, as it relies on the initial definition of *W*_0_, that is, fixing the amine|mica binding energy (and hence *k*^A^ values) using this value. Therefore, we reduce the free parameters from four rate constants to two, resulting in a simple two parameter estimation for the equilibrium constants for the interfacial ion interactions *k*^I^. As a word of caution, this pins the numeric values estimated for the equilibrium constants in the model to the thereby chosen set of fixed amine interaction parameters.

On the basis of this choice, Figure 4B visualizes the 2-dimensional parameter variation of ion exchange rate constants *k*_on_^I^ versus *k*_off_^I^ in terms of the RMSD. As can be seen in the plot, we find a minimum of the RMSD in a specific broad area, where we obtain the point indicated by a red cross as the optimized parameter choice. The numeric values for the ion-exchange rate constants are hence estimated as
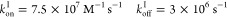
1resulting in a p*K*_I_ = 1.39 and a corresponding interaction free energy Δ*G*_I_ = 3.21 kT. This estimated p*K*_I_ agrees well with literature results.^49^ Further, the interaction free energy is in the range expected for a Coulomb interaction;  of the free energy for two opposite charges interacting across water at a separation, *r*, of half a nanometer.^40^ It is worth noting that the minimum is rather shallow, and points in the minimum region all yield rather similar thermodynamics, with slightly varying kinetic parameters.

As such, the free energies obtained from our model hence suggest that Δ*G*_I_ < Δ*G*_A_, which implies that the ion-to-mica binding energy is weaker than the amine-to-mica bond. On the other hand, the *k*_on_ of both species are at the same order of magnitude, suggesting an effective competition for a binding site with both species having similar bind frequencies at steady-state. Further, the dissociation rate constants suggest that the unbinding for ions is considerably faster than for amines, consistent with the interaction free energy difference. As a result, at low ion concentrations the amine|mica bond overpowers the ion–mica binding, whereas the ions “flood” the mica lattice at high concentrations, explaining the observed decay of the adhesion as a function of the electrolyte concentration.

Coming back to the deviation of the simulation between 0.05 and 0.2 M. This bias is not related to the rate constants, but rather to the simplified description of the adhesive interface. Specifically, the boundary conditions include the definition of a fixed interaction volume *V*_s_ at the adhesive interface, which was fixed to 3 nm height with unit area (based on the force versus distance characteristics, see Methods and Materials section). Further, the model currently estimates the interfacial ion concentration based on the bulk concentration. However, the formation of an electric double layer, will lead to effectively higher interfacial concentrations, in particular at lower bulk concentrations at a highly charged interface, which in turn lowers the measured amine-binding more significantly than in the model. Including an estimated interfacial concentration from electric double layer models is beyond the scope of this work. As such, at low concentrations we estimate less ions facing the mica, facilitating the amine|mica bonds and enhancing the simulated over the measured adhesion. Experiments and simulations converge for *C* > 0.2 M, at which the abundance of ions overpowers the effect of this bias.

In summary, our model catches the essence of the observed competition, with thermodynamic values that compare well with simulation and other experimental data. Further work is necessary in order to properly include an effective interface concentration (e.g., an interfacial activity), and all those effects related to amine protonation based on the solution pH, which would strengthen the model, as well as to complement this work with independent single molecule measurements that would further confirm the rate constants, for example, via Bell-Evans (*k*_off_) analysis.^48,50^ Further, and as shown in the inset in Figure 4A the model allows estimation of the binding density *B*/*A*, that is, the ratio between the total number of bonds formed *B* (bound amines) to amines available at the interface. On the basis of the simulated ratio we can estimate that the number of formed amine bonds decreases from 80% to 10%, from 0.01 to 1 M, providing us a detailed insight into the concentration dependent molecular bond distribution in the adhesive contact.

**Figure 5:**
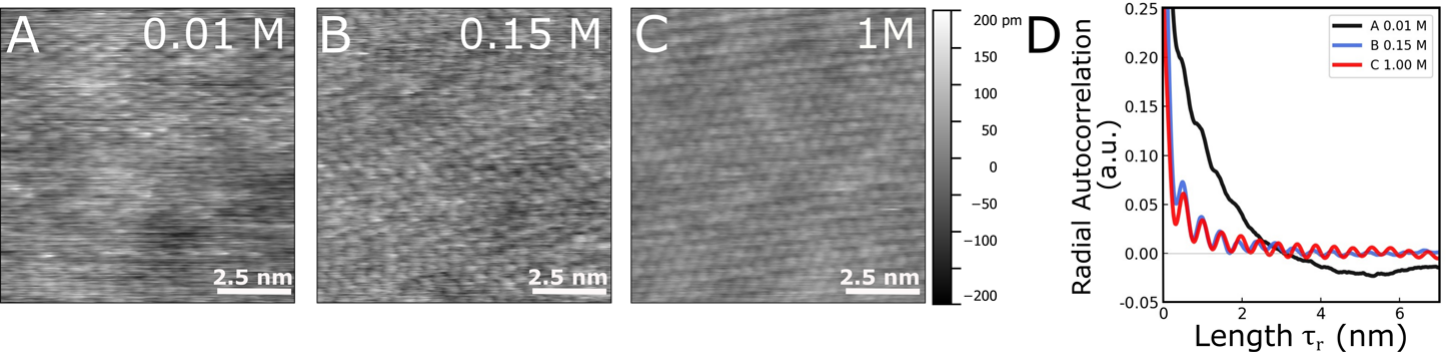
Molecularly resolved AFM topography (10 × 10 nm^2^) on mica samples for respectively 0.01 M (A), 0.15 M (B), and 1 M (C) sodium chloride concentration. (D) Radial autocorrelation for the three conditions. The periodicity is well-defined at higher electrolyte concentration, confirming the presence of a more and more ordered cation layer at higher concentrations.

We now complementarily visualize the increasing ion occupancy at the mica binding sites using super-resolved *in situ* AFM imaging. Figure 5 shows AFM topographies acquired in amplitude modulation mode (AM-AFM) on a freshly cleaved mica surface in 0.01 M (A), 0.15 M (B), and 1 M (C) sodium chloride solutions. All three images show a 10 by 10 nm^2^ area with the same color bar scaling. All images indicate highly resolved ion adsorption at the mica interface. Yet, a clear trend is observed in which the surface structure becomes more ordered and defect-free when going from low to high concentration. Qualitatively, this can be seen from the decreased contrast of the images at higher concentration. Further, and as shown in Figure 5D this trend is confirmed by a quantitative post analysis of the radial auto correlation function, which reveals a clear increase in long-range periodicity with more ions present in the solution.

As a side note, for highly resolved imaging in solution it is generally not straightforward to interpret the obtained images at a molecular level. This occurs largely because a molecular structure in solution is, in essence, an equilibrium of adsorption and desorption at steady state. The fitting result from our measurements above, however, allows us some further insight. Specifically, the interaction free energy of ions with mica is in the range of a Coulomb interaction at a distance of half a nanometer. Considering the hydrated sodium ion radius (0.4 nm)^40^ this suggests that the imaged ions in the lattice are adsorbed strongly, however with their hydration shells intact. This is an interesting outcome, and suggests that our complementary approach will also prove useful for arriving at molecular level interface science, where highly resolved imaging data can be interpreted in more detail, and in direct comparison to theoretical modeling in future work.

## Conclusions

We utilized a model system with full control of the polymer density ρ, allowing us to unravel the competition between amines and cations for the negatively charged surface binding sites on mica. In addition, we also assessed the effect of an increase of the lateral amine density. At the transition from the mushroom to the brush regime at 5% coverage the amine|mica interaction shows a peak of the adhesion force due to entropic and background effects. Increase of the electrolyte concentration resulted in an exponential decrease of the adhesion force at all coverages. A kinetic model based on two competing Langmuir isotherms, one for the ion adsorption and one for the amine|mica bond formation, describes this exponential decay well. Small subtleties and deviations of the simulated and measured data were related to limitations of the model. In particular, the relation of the equilibrated interfacial ion concentration in the adhesive contact, and the bulk concentration will be included into the model in future work. The very simple model still catches the essentials and the concentration dependent behavior very well. The presented experimental setup offers an ideal model system for further experimental and theoretical studies of competitive adhesive interactions of increasingly complex systems.

## Methods and Materials

### Materials

Milli-Q water (Milli-pore, TOC value < 2 ppb, resistivity > 18 MΩcm) is used throughout. The lipids used are 1,2-distearoyl-*sn*-glycero-3-phosphoethanolamine (DSPE) and 1,2-distearoyl-*sn*-glycero-3-phosphoethanolamine-N-[amino(polyethylene glycol)-2000] (ammonium salt) (DSPE-PEG_amine_), purchased from Avanti Polar Lipids. The DSPE is dissolved in a mixture of 70% chloroform min. 99.9% Rotisolv from Carl Roth, 30% methanol from J.T. Baker, and three drops of Milli-Q water (per 100 mL of total solution) to ensure a well diluted solution at *C*_DSPE_ = 0.25 mg/mL. DSPE-PEG_amine_ is solely dissolved in chloroform, at *C*_DSPE–PEG_ = 0.1 mg/mL.

The following chemicals were used: sodium chloride (min 99% purity) from Carl Roth, *n*-hexane min. 98% from Carl Roth, ethanol absolute (min 99.9%) from VWR, 1-hexadecanethiol 99% (C_16_) from Sigma-Aldrich, Inc. During sample preparation, EPO-TEK heat curable glue (EPO-TEK 377) from Epoxy Technology and UV curable glue (NOA 81) from Norland Products Inc. were used.

### Surface Forces Apparatus (SFA)

The SFA is an optical technique based on multiple beam interferometry between two semireflecting mirrors.^51^ It can measure the distance between the mirrors with subnanometer resolution and the interaction force, obtained from an independent force sensor, has a detection limit of ≃0.1 μN/m.^52^

Figure 1A shows a schematic representation of the home-built SFA used.^52^ Starting from the top of the panel, we have an asymmetric configuration where one mirror (gold) is used as a substrate for the lipid model system (LMS) and apposing it is a back-silvered mica surface. The silver (35 nm) was deposited using physical vapor deposition (PVD) at a pressure of 1.7 × 10^–6^ mbar (system built at TU Wien). The mica layer is secured onto a cylindrical quartz disk (from SurForce LLC.), with a radius of curvature *R*_C_ = 0.02 m, by applying UV cured NOA 81 glue.

The muscovite mica used has 3 to 6 μm thickness and a nominal unit cell area *a*·*b* = 46.6 Å^2^.^53^ The atomic coordinate of the potassium ion in the mica crystal is located at the center of hexagonal structures built up from silica tetrahedra. Peeling-off a mica layer generates negative binding sites, when the potassium ions dissolve in aqueous solutions. Thus, we can define the area per binding site as Σ_mica_ = 46.6 Å^2^/site.

The light from the optical cavity, defined by the mirrors, produces an interference pattern (Newton’s rings) and fringes of equal chromatic order (FECO), via a diffraction grating, which are projected onto a 2D detector in the spectrometer^51^ (see Figure 1A and previous work for more details^54^).

The used setup is suspended by a bungee chords mechanism, which damps external and building vibrations. One of the surfaces is mounted on an adapter connected to a highly sensitive strain gauge providing online information about the forces directly, during the experiment. In addition, since the force does not have to be obtained from the optical information at all, we can compose force–distance curves in both force and distance are independent variables.^11,52^ Further, FECO are analyzed with the SFA Explorer, a previously developed software package, capable of returning the distance between the mirrors by fitting the thicknesses of all the layers forming the optical cavity.^54^

### Atomic Force Microscopy (AFM)

We use a Cypher ES (Asylum Research, Oxford Instruments, Santa Barbara, CA) to acquire super-resolved images of a mica layer immersed in sodium chloride solutions. The mica layer is freshly cleaved and glued on a magnetic disk using UV cured NOA 81 glue.^11^ Imaging is performed in amplitude modulation mode driven by blueDrive photothermal excitation (laser power 9 mW) and using reflex gold coated, ultra high frequency, silica probes (ARROW-UHFAuD, NanoWorld, Switzerland).

Images of the topography are recorded over a scan area of 10 by 10 nm^2^ or 20 by 20 nm^2^ with 256 or 512 points and lines. The scan rate and set point are varied within the range of 6.5–8 Hz and 15–70 mV to optimize image quality for the various salt concentrations. AFM data analysis is performed with Gwyddion 2.55 and Python 3.8.

### The Lipid Model System

Figure 1B presents the model system used in this work. This system is experimentally built as follows: First, we deposit 35 nm of gold onto freshly cleaved mica layers by PVD at 1.7 × 10^–6^ mbar. Then, EpoTek glue 377 (heat cured 2 h at 150 °C) is used to glue the gold side of the layer (top side down) onto an SFA disk with a nominal radius of curvature *R*_C_ = 0.02 m. Following slow cooling to room temperature, we mechanically remove the mica layer, under ethanol, to expose the atomically smooth gold substrate. After this step the gold surface is not allowed to dry, to minimize airborne contamination during the next step.

Afterward, the disk is immersed in the thiol solution (0.5 mg/mL C_16_ in ethanol filtered with a 0.2 μm pore size filter) for 1.5 h in a dark environment. Subsequently, it is immersed for 10 seconds in *n*-hexane and in a bath of filtered ethanol thereafter. The sample is then dried in a stream of nitrogen and placed in the SFA holder. This process ensures an inner layer based on strong thiol anchoring onto a templated ultrasmooth gold, which enhances the stability of the model system.^11^

Finally, a mixture of DSPE and DSPE-PEG_amine_ is deposited on the hydrophobized surface using a Langmuir–Blodgett trough (LBT). A mixture of these two lipids forms the outer leaflet of the model system. The ratio of the mixture is controlled by the target polymer coverage, Γ, defined as follows:
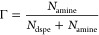
2where *N*_dspe_ and *N*_amine_ are the number of molecules for DSPE and DSPE-PEG_amine_, respectively. Controlling the ratio of DSPE and DSPE-PEG_amine_ allows us to carefully and reproducibly control the density of amines at the interface, indicated by ρ in the text.

LBT depositions are generally performed at a lateral pressure of 40 ± 1 mN/m, to deposit a gel-phase lipid layer with limited lateral diffusion. On the basis of the LBT measurements we can also determine the area occupied by one molecule of the lipid mixture .

Afterward, by immersing our *C*_16_ coated surface (speed of the vertical translator 15 μm/s), we deposit a carefully decorated outer lipid monolayer for direct force versus distance probing in the SFA. After LBT deposition, samples are not allowed to dry again, and are kept under water at all times.

### Simulation of the Nanoscopic Competition

The competition taking place in the experimental system concerns three interacting species; the polymers terminated with amines (), the ions () and the mica binding sites (). A Langmuir adsorption isotherm (LAI) can describe the interaction of each species with the interfacial binding site. Hence, we can interpret the interfacial interaction as two competing isotherms, one for the ion adsorbing on the mica surface () and a second one for the amine|mica bond formation (). Consequently, the equilibrium between these five populations (, , ,  and ) can be expressed in terms of the following chemical reaction:
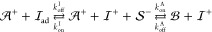
3where the labels A and I stand for amines and ions, respectively. With *k*_on_ we indicate the rate constant of producing  or , and with *k*_*off*_ we indicate the rate constant of the inverse process. For each isotherm we define the equilibrium constants  and , which are related to the variation of free energy by
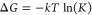
4We can further express each species of eq 3 in an interacting adhesive contact as a number of molecules per area ( as *x*_1_,  as *x*_2_,  as *x*_3_) or per effective interaction volume *V*_*s*_ ( as *x*_4_,  as *x*_5_). Here, the effective interaction volume is chosen as 3 nm times the unit area, which is consistent with the observed distance changes during breaking of an adhesive contact.

By evolving these concentrations in time, we can define a set of ordinary differential equations (ODEs) expressing the set of chemical reactions (eq 3) of the competing Langmuir isotherm model as follows:
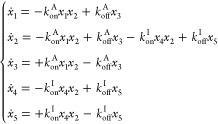
5

We numerically solve set of eqs 5 with a Python 3.8 script using the Runge–Kutta method of order 4 (with time steps at order 5 accuracy) as implemented in the SciPy library.^55^

The ODEs describing the competing equilibria are solved by setting the equilibrium constant of the amine|mica interaction to experimentally obtained values, in terms of the interaction free energy. Specifically, from the SFA experimental data, we can estimate the interaction free energy (work of adhesion) per polymer *W*_0_ (which is an upper bound for the amine|mica energy). Thus, inverting eq 4 for amines, we can further estimate the amine equilibrium constant from SFA measurements . Consequently, we fix the amine rate constants to the exerimental findings, leaving the rate constants related to ions (*k*_on_^I^ and *k*_off_^I^), as the only fittable free parameters. Finally, the latter are varied to obtain the best agreement between experimental and simulated data in terms of a linear least-squares optimization (visualized by the root-mean-square deviation, RMSD).
